# The effect of land use change on soil fertility parameters in densely populated areas of Kenya

**DOI:** 10.1016/j.geoderma.2019.02.033

**Published:** 2019-06-01

**Authors:** Daniel Kyalo Willy, Milu Muyanga, Joseph Mbuvi, Thomas Jayne

**Affiliations:** a Kenyatta University, Department of Agribusiness Management and Trade, PO BOX 43844, Nairobi, Kenya; b The African Agricultural Technology Foundation, Nairobi, Kenya; c University of Nairobi, Department of Land Resource Management and Agricultural Technology, PO Box 30197, 00100 Nairobi, Kenya; d Department of Agricultural, Food and Resource Economics, Michigan State University, East Lansing, MI 48824, USA

**Keywords:** Agricultural intensification, Soil quality, Land use change, Sustainable

## Abstract

The current study seeks to assess sustainability of agricultural land use by identifying the effect of land use change on soil quality using cross-sectional data collected through a household survey among 525 farm households in densely populated areas of Kenya. Soil samples were collected, analyzed and compared across three land use types: undisturbed, semi-disturbed and cultivated. To achieve these objectives, descriptive, Nutrient Index approach and Classification and Regression Tree (CART) analysis methods were used. Results indicate that within a period of five decades, agricultural land use has led to a decline in Total Organic Carbon (−72%), Magnesium (−65%) and Boron (−61%), Iron (−22%) and Total Nitrogen (−15%). The drivers of deterioration identified were cutting across inherent properties such as soil chemical (pH), physical (soil mapping unit) and biological (organic carbon) attributes, farmer practices (agricultural commercialization) and exogenous factors (population density and Agro-ecological zones). The study concludes that indeed conversion of land from natural vegetation is associated with deterioration in soil quality and therefore policy needs to create incentives for the build-up of soil organic matter, replenishment of soil macro and micro nutrients. Blending of commercial fertilizers with targeted micro-nutrients, maintenance of soil conservation techniques and long term fallowing are encouraged.

## Highlights

• Agricultural land use has led to decline in OC, Mg, B and total N.
• Deterioration driven by inherent soil properties, farmer practices and exogenous factor
• Policy action: increasing soil organic matter and replenish macro and micro nutrients
• Mandatory blending of commercial fertilizers with micro-nutrients desirable

## 1 Introduction

The sustainable Development Goals (SDGs) aim to end poverty, protect the planet and ensure prosperity for all by 2030 among other aspirations (UN General Assembly, 2015). Improved agricultural productivity has a great role to play in poverty eradication and reduction of hunger among millions of people living in poverty globally (World Bank, 2008). However, any form of land resource degradation can greatly undermine agricultural productivity and reduce the possibility for achieving these noble goals. Recent studies conducted in different parts of Kenya have painted a picture of declining crop productivity, a trend that has been attributed to factors such as population density growth (Muyanga and Jayne, 2014) and continuous cultivation without addition of external inputs (Mugendi et al., 2007). Previously, sustainable forms of agricultural intensification were reported even when population density was growing (Mortimore et al., 1991; Mortimore and Tiffen, 1994; Tiffen et al., 1994; Zaal and Oostendorp, 2002). However, it has been noted that at higher population densities, the gains associated with sustainable agricultural intensification can be reversed as a result of land management practices that undermine soil conservation (Willy et al., 2019). Land use changes have particularly been associated with changes in soil parameters. Don and Schumacher (2011) indicate that when tropical cropland is converted to grassland or fallow, Soil Organic Carbon (SOC) stocks within the 40 cm depth and 20 cm depth can increase by 25% and 32.2% respectively. In comparison to the changing land use, global SOC stocks within 15 cm depth at sites without any land use change have reportedly increased by 0.19% C y^−1^ over recent decades (Chen et al., 2015). This clearly shows the land use change can have effects on soil fertility as also confirmed by more recent studies (e.g. Rolando et al., 2018). Further, agricultural land use may also interfere with the biological components of the soil through the effect of tillage and the use of fertilizers and other agro-chemicals on soil microorganisms. Gómez et al., 1999 indicate that continuous conventional tillage can have negative effects on soil physical attributes such as bulk density and texture, influencing critical functions such as water infiltration. Agricultural land use may in itself not be entirely detrimental to the environment provided that farmers implement sustainable land management practices such as consistent addition of organic matter, soil and water conservation practices, crop rotation and fallowing. However, the quality of agricultural land can be compromised through practices such as continuous cultivation without fallowing leading to nutrient mining, use of the same types of acidifying fertilizers without liming and dis-adoption of soil conservation practices in response to population driven land pressures (Willy et al., 2019). Given that the consequences of deterioration in soil fertility associated with land use change are dire, it is important to understand which specific soil quality parameters are affected by agricultural land use and which agricultural practices or environmental factors are responsible for the greatest decline in critical soil quality parameters. Although it is important to assess how agricultural land use influences specific soil fertility parameters, we find a dearth of knowledge on how soil fertility parameters change with land use change and farming practices. Against this backdrop, the current study sought to; (1) assess the soil physical, chemical and biological status in three land use types in the densely populated areas of Machakos and Makueni Counties (2) assess the effect of cultivation and long term fallowing on the level of soil chemical, physical and biological parameters in Machakos and Makueni counties; (3) Identify the factors influencing the changes in selected deficient/deteriorated soil quality parameters. This study adds to the body of knowledge through its unique approach where the spatial analogue approach is used to compare soil parameters of soil samples drawn from cultivated fields with those drawn from parcels in their pristine conditions to show how cultivation has impacted on soil quality. Through the use of CART, the current study goes beyond the conventional approach where studies simply look at how crop production has impacted soil fertility at very general level. On the contrary, the current study goes a step further to look at how cultivation has impacted on specific soil fertility parameters while focusing on both endogenous and exogenous drivers of soil parameter change. This approach and the results obtained thereof can be widely replicated in areas with similar agro-ecological conditions when modeling the influence of crop production of soil fertility parameters.

## 2 Methodology

### 2.1 Description of study areas

The current study was carried out in Machakos and Makueni Counties of Kenya. Six study sites (Kangundo, Iveti, Mbooni, Kilungu, Kalama and Wote) were selected from the two counties to capture differences in population density, soil mapping units and Agro-Ecological Zones. Given their differences in geomorphological, topological and climatic characteristics, it is expected that effects of agricultural land use on soil fertility may differ across the sites. These sites are summarized in Table 1.

**Table 1:** Description of the study areas.

County	Region	AEZ^a^	Mean population density (persons/km^2^)	Soil mapping units	Mean altitude (m.a.s.l.)	Average rainfall (mm/annum)
Machakos	Kanzalu	III	964	HNr-1	1696	800–1200
	Iveti	III	633	HNr-1	1889	800–1200
	Kalama	III	200	UNr-1	1482	800–1200
Makueni	Kilungu	II/III	451	MQ1	1833	1200–1600
	Mbooni	II/III	527	MQ1	1811	1200–1600
	Wote	IV	148	UQ1	1180	400–800

a The Agro ecological zones represent areas of similar potentials and constraints for development. The study area has been categorized into the following zones: II = high potential; III = medium potential IV = semi-arid for more details see FAO (1996).

Kangundo is located in the western slope of Kanzalu range within AEZ III characterized as medium to high potential. The area is located at an altitude ranging between 1500 and 1800 m and receives bimodal rainfall averaging between 800 and 1200 mm annually. This site was located within HNr1 soil mapping unit, which consists of soils that developed on banded gneisses, biotite garnet gneisses and tuff, well drained, moderately deep to very deep, dusky red; friable clays, Rhodic Nitisols: FAO (1998): Very fine, mixed, isothermic Oxic Paleustalf: Soil Taxonomy. These soils are characterized by moderate Carbon (C) content, low Nitrogen (N), slightly acid to neutral pH, deficient Phosphorous (P) and medium Cation Exchange Capacity (CEC). Crop production in the area is dominated by coffee, maize, beans, bananas and a variety of vegetables.

The Iveti site is located in the Iveti Hills, approximately 15 km from Machakos town to the East. A larger part of the hill is covered by a government owned forest which is surrounded by private settlements. Soils in the area are also in the HNr1 as described above. Majority of the farmers grow coffee with an average farm size of 0.5 ha where zero grazing dairy production is also popular. Further, farmers in Iveti engage substantially in farm forestry, dominated by Eucalyptus (*Eucalyptus globulus*), Wattle (*Acacia pycnantha*), Grevillea (*Grevillea robusta*), and Avocado (*Persea americana*) fruit trees. The Kalama site is located on the Kalama uplands on the Western side of Machakos Town AEZIII. Soils in the area belong to the UNr1 mapping unit consisting of soils that developed on biotite and banded gneisses, well drained, deep to very deep, dark reddish brown to dark red, friable clay and sandy clay, Haplic Ferralsol; FAO (1998): Clayey, kaolinitic, isothermic, Typic Haplustox Soil Taxonomy. The soils are characterized by low levels of CEC, organic carbon, Phosphorous and Nitrogen. These soils were mainly found in the Kalama uplands of Machakos County. The Mbooni site is situated in the Mbooni hills AEZII/III and also Kilungu site in Kilungu hills within Makueni County located at an altitude of >1800 m.a.s.l. are receiving rainfall ranging between 1200 and 1600 mm per annum. Soils in the area fall under the MQ1 soil mapping unit, consisting of soils that developed from predominantly granitoid and quartzofeldspathic gneisses, well drained, moderately deep to very deep, brown friable sandy clay to clay; Humic Cambisols FAO (1998): Fine, mixed, isothermic Ustoxic Humitropets: Soil Taxonomy. These soils are characterized by pH which is extremely acid to very acid; deficient phosphorus (P); medium Cation Exchange Capacity (CEC); adequate Carbon (C) and low to medium Nitrogen (N). Dominant crops include coffee, maize, beans and a wide range of vegetables. Finally, Wote study site is located near Wote town which lies in AEZ IV characterized as semi-arid. Soils in the area belong to the UQ1 mapping unit consisting of soils that developed on gneisses, well drained, moderately deep to deep, dark yellowish brown to dark brown, friable to firm gravelly sandy clay loam to clay with top soil of loamy sand to sandy loam, Ferric Luvisols: FAO (1998): Coarse loamy, mixed, isothermic Oxic Haptustalf Soil Taxonomy. These soils are characterized by low organic carbon, are sufficient in P and the CEC is low. The elevation ranges between 750 and 1250 m.a.s.l. and being a semi-arid area, receives rainfall between 400 and 800 mm per annum. Drought resistant crops such as sorghum and millet are also predominantly grown in the low lands of Makueni where extensive livestock production is a key economic activity. Some areas also engage in cotton production, although the industry has been stagnating owing to dwindling markets. Farmers in Makueni also produce Mangoes which are targeted for the urban markets.

### 2.2 Sampling and data collection methods

The current study used a spatial analogue approach whereby soil fertility parameters were compared across parcels located in three management regimes: un-disturbed, semi-disturbed and cultivated. The undisturbed parcels were those that had not been used for cultivation or any other intensive use (such as intensive grazing) for >50 years and were in the pristine status covered by indigenous vegetation. Parcels were categorized as semi-disturbed if they had previously been used for cultivation but at least not within the last 10 years preceding the study. Parcels that had never been used for cultivation but were highly disturbed, probably through intensive grazing also fell under this category. The last category included parcels that were used for cultivation for a period exceeding 30 years prior to the study. The most common crops that had been grown in these fields similar to those described in Section 2.1.

A multi-stage sampling approach was used in the study. In the first stage, undisturbed parcels of land were identified through the snow-balling procedure with the help of elderly villagers. Snow balling is a non-probabilistic sampling technique which involves the identification of the first subject (in this case an undisturbed parcel of land) and then using referrals, identify the next subjects until the contact persons cannot identify any more. In the entire research area, 37 undisturbed parcels were identified. In the next stage, a sampling frame was developed where all the households located close to the undisturbed parcels were listed and then a random sample drawn. From each sampled household, the largest maize plot in the 2015/2016 long rains season was identified for inclusion in the study under the cultivated category. A final validation of the selected plots was done to ensure that the plot was within close proximity to the undisturbed plot (mean distance: 360 m) and that the plot was within the same soil mapping unit as the undisturbed plot. Plots that did not meet the selection criteria were replaced. Finally, at least one semi-disturbed plot was identified close to each undisturbed plot.

From each sampled household, socio-economic data relating to land use, crop production trends and other important social demographic and economic aspects were collected through a household survey. The survey involved administration of a semi-structured interview schedule. Further, from each selected parcel in the three categories soil samples were collected. In every parcel five sampling points were identified using a zig-zag layout. From each sampling point, a sample was drawn using a soil auger within a depth of 15 cm. The five samples were then mixed in a bucket and composite sample weighing approximately 250 g drawn from the mixture and packed in a zip-loc bag. The samples were then properly labeled and later delivered to the CropNuts soil laboratory in Nairobi for further processing and analysis.

### 2.3 Soil and data analysis methods

In the soil laboratory, soil samples were air dried, carefully pounded with pestle and mortar and passed through a 2 mm sieve. Organic carbon was determined according to the Nelson and Sommers method (Nelson and Sommers, 1986) while total Nitrogen was determined using the Bremner and Mulvaney (1982) method. The Mehlich III extraction method was used to determine exchangeable bases, micronutrients and available P. This is because it allowed analysis of multiple elements from the extractant using inductively-couple plasma spectroscopy (ICP) (Mehlich, 1984). Soil pH and EC were determined in 1:1 water suspension. Reactive carbon was determined using the calorimetric method following Weil et al. (2003). To determine the soil texture, samples were first pre-treated in order to remove organic matter, salts and any other cementing elements present (Bowman and Hutka, 2002) followed by fractionation, drying and weighing of the sand, silt and clay.

To assess the soil chemical, physical and biological attributes, descriptive statistics were used where the mean and standard deviation of key macro and micro nutrients (N, P, K, Mg, Cu, B, Zn); soil chemical properties, (pH, EC, CEC), biological attributes (Organic Carbon, Reactive Carbon) and physical characteristics (texture) were computed. The values were compared across land use types while checking statistical significance in mean differences using analysis of variance (ANOVA) while the homogeneity of variances was done using the Levene's test in SPSS 24.

To assess the effect of cultivation and long term fallowing on soil fertility parameters two approaches were used. First, the percentage changes in the individual soil fertility parameters as land use changes from undisturbed to cultivation and from cultivation to fallowing were computed to capture declines or improvements in the parameters. Secondly, a Nutrient Index (NI) was computed for each parameter following the Ramamurthy and Bajaj (1969) approach. A nutrient index is an estimate of the % distribution of soil samples across three categories: low, medium and high classes of nutrient status as per the soil test interpretation guide by Horneck et al. (2011). The Nutrient index was computed using Eq. (1):

(1) $$\mathrm{NI}=\left(\left(N_{L}*1\right)+\left(N_{M}*2\right)+\left(N_{H}*3\right)\right)/N_{T}$$

where *N*_*L*_, *N*_*M*_ and *N*_*H*_ indicated the number of samples falling in the low, medium and High classes of nutrient status and *N*_*T*_ is the total number of samples. Levels of the nutrient index are then calculated as per the following guide: Low (<1.67); Medium (1.67–2.33) and High (>2.33). The effect of land use change on soil quality was then assessed by comparing the Nutrient Index levels across the land use types.

From the first and second objective, the soil parameters that were deficient and/or had deteriorated as a result of cultivation were identified. For such parameters, a follow up was done to identify the factors that could explain their variability. This was achieved using the Classification and Regression Tree Analysis (CART) method in SPSS version 24. CART analysis (Breiman et al., 1984) is a non-parametric method used to select explanatory variables that are important in determining the outcome variable from a large number of variables (Table 2). It identifies the important independent (predictor) variables that explain the variation in a dependent (target) variable. The predictor variables as presented in Table 2 included endogenous plot level characteristics (farm size, location, slope and soil erosion status); biophysical characteristics (soil type, altitude and agro-ecological zone); soil conservation practices implemented on the plot (number of practices implemented per ha); lagged soil fertility management (amount of fertilizer and manure applied in previous years) and household socio-demographic; economic attributes (age, gender, household size, land size, wealth index, commercialization index) and population density.

**Table 2:** Dependent and Independent variables used in the CART analysis.

Variable	Variable description	Unit of measure
Dependent variables		
C	Total organic carbon	%
P	Plant available phosphorus	ppm
N	Total nitrogen	%
B	Boron	ppm
Cu	Copper	ppm

Explanatory variables		
Regional fixed effects		
POPDENS	Sub-location level population density	Persons/km^2^
AEZII/III	Plot located in agro-ecological zone III	Dummy (Yes = 1)
AEZIV	Plot located in agro-ecological zone IV	Dummy (Yes = 1)
SMU1	Soil mapping unit = MQ1	Dummy (Yes = 1)
SMU2	Soil mapping unit = HNr1	Dummy (Yes = 1)
SMU3	Soil mapping unit = UQ1	Dummy (Yes = 1)
SMU4	Soil mapping unit = UNr1	Dummy (Yes = 1)
Plot attributes		
LOWERO	Extent of soil erosion in the plot is low	Dummy (Yes = 1)
MODERO	Extent of soil erosion in the plot is moderate	Dummy (Yes = 1)
HIGHERO	Extent of soil erosion in the plot is high	Dummy (Yes = 1)
EXSLOPE	Farm where plot is located is very steep	Dummy (Yes = 1)
MODSLOPE	Farm where plot is located is hilly	Dummy (Yes = 1)
FLATSLOPE	Farm where plot is located is undulating	Dummy (Yes = 1)
PLOTHA	Size of the plot	ha
CCROP	Cash crop planted on the plot	Dummy (Yes = 1)
SCEXT	The extent of soil conservation^a^	
Land management practices		
FERTHIST	No. of years of consistent fertilizer application	Years
MANHIST	No. of years of consistent manure application	Years
FERTQTY	Quantity of inorganic fertilizer applied	kg/ha
MANQTY	Quantity of organic fertilizer applied	kg/ha
Institutional factors		
EXTACC	Distance to extension service providers	km
MKTACC	Distance to nearest markets	km
INFACC	Distance to tarmac road	km
Soil attributes		
pH	Soil pH	Number
CLAY	Soil clay content	%
SAND	Soil sand content	%
CEC	Cation Exchange Capacity	Number
C	Total organic carbon	%
ROC	Reactive organic carbon	%
Household characteristics		
ADULT	Number of adults in the household	Number
AGE	Age of household head	Years
EDUCLEV	Average education level of the household	Years
INCOME	Annual total household income	Ksh.

a The soil conservation intensity index was calculated incorporating the number of soil conservation practices and the duration within which farmers had implemented the practices.

The rationale for the choice of the explanatory variables is based on the pretext that soil fertility is determined first by endogenous plot level attributes and also exogenous factors related to the farmer and other regional fixed effects. Further, the dynamics in soil fertility depend more on historical practices such as soil and water conservation and fertility management practices (Gicheru et al., 2004). The performance of the CART model was done using the splitting procedure through the Gini index, one of the most popular split selection methods. This was used for impurity measure and goodness-of-fit measure in the classification procedure. Further, the Hosmer-Lemeshow goodness of fit test was used to confirm the suitability of the trees.

## 3 Results and discussions

### 3.1 Characteristics of soils in the sampled plots

Table 3 presents the descriptive statistics on the soil chemical, physical and biological attributes which are compared across the three land use types. Soil pH ranged from very strongly acidic to moderately alkaline. The mean pH was 6.0 and 6.1 in the control and cultivated plots respectively. The mean pH in the cultivated plots was in the suitable range. However, about 47.3% of the samples had pH levels in the classes moderately acid (24.2%), strongly acidic (19.7%) and very strongly acidic (3.4%). Such pH levels can substantially affect the availability of nutrients through its effect on soil microbial activity (Aciego Pietri and Brookes, 2008).

**Table 3:** Descriptive statistics on the effect of land use on soil properties.

	Undisturbed (U) n = 37		Semidisturbed (S) n = 45		Cultivated (C) n = 350		p-Value	%∆ U to C^1^	%∆ C to S^2^
	Mean	SD	Mean	SD	Mean	SD		(C − U/C) ∗ 100	(S − C/S) ∗ 100
pH	6.0^a^	0.61	5.8^b^	0.69	6.1^a^	0.72	0.03^⁎^	1.8	−5.0
EC (μS/cm)	77.4^a^	46.74	43.1^b^	23.08	54.3^c^	29.14	0.0001^⁎⁎^	−42.5	−25.9
P (ppm)	8.5^a^	7.01	7.1^a^	5.71	31.6^c^	36.04	0.0001^⁎⁎^	73.0	−346.0
K (ppm)	220.0	183.47	184.8^b^	139.05	283.7^c^	143.34	0.0002^⁎⁎^	22.4	−53.5
Ca (ppm)	1233.9^a^	585.81	749.1^b^	576.71	914.0^c^	417.81	0.0002^⁎⁎^	−35.0	−22.0
Mg (ppm)	377.9^a^	215.36	235.8^b^	178.41	229.3^c^	113.30	0.00001^⁎⁎^	−64.8	2.7
S (ppm)	10.6^a^	5.70	11.8^a^	7.22	12.3^b^	7.85	0.343^ns^	13.8	−4.3
Na (ppm)	30.6^a^	9.29	30.5^a^	13.23	27.9^b^	12.49	0.126^ns^	−9.6	8.5
Fe (ppm)	133.5^a^	46.16	118.6^b^	47.63	109.4^c^	40.38	0.0001^⁎⁎^	−22.0	7.7
Mn (ppm)	100.8^a^	82.38	67.0^b^	53.29	104.1^c^	84.78	0.024^⁎^	3.2	−55.3
B (ppm)	0.8^a^	0.45	0.4^b^	0.18	0.5^c^	0.26	0.00001^⁎⁎^	−60.8	−34.8
Cu (ppm)	2.0^a^	0.77	2.1^a^	1.35	4.3^b^	5.44	0.021^⁎⁎^	52.8	−109.1
Zn (ppm)	3.6^a^	2.26	3.4^b^	3.39	6.8^c^	10.86	0.046^⁎^	46.2	−99.1
CEC (meq/100 g)	12.9^a^	4.74	8.3^b^	4.35	9.4^c^	3.06	0.0001^⁎⁎^	−37.1	−13.5
Total N (%)	0.30^a^	0.05	0.15^b^	0.06	0.11^c^	0.05	0.050^⁎^	−15.0	7.4
C/N	16.1^a^	2.32	14.2^b^	2.64	13.7^c^	2.62	0.00023^⁎⁎^	−17.3	3.6
Sand (%)	61.1^a^	11.40	59.2^a^	12.39	58.0^a^	12.06	0.562^ns^	−5.5	2.1
Silt (%)	9.7^a^	3.23	8.6^b^	3.39	7.5^c^	3.51	0.0002^⁎⁎^	−28.4	12.4
Clay (%)	29.2^a^	10.21	32.2^b^	11.71	34.5^c^	11.38	0.034^⁎^	15.4	−7.1
TOC	3.0^a^	1.03	2.1^b^	0.94	1.7^c^	0.84	0.00001^⁎⁎^	−72.1	15.6
Reactive carbon (%)	0.13^a^	0.03	0.07^b^	0.03	0.05^c^	0.02	0.00000^⁎⁎^	−58.8	11.2

* & ** statistics are significant at the 0.05 and 0.01 levels respectively.

Means sharing the same letter along the row are not statistically significant.

CEC = Cation Exchange Capacity: TOC = Total Organic Carbon; EC = Electrical Conductivity.

1 This is the percentage change in soil nutrient as a result of conversion of land to crop land.

2 This is the percentage change in soil nutrients as cropped land is left fallow for a long period (>20 years).

The mean Total Nitrogen in the cultivated parcels was 0.11%, ranging between 0.04 and 0.22 which was much lower than the levels found in the undisturbed plots which averaged at 0.30%. Total Nitrogen was generally deficient in most of the soil samples, especially in the cultivated plots, considering that the level considered adequate is 0.2% (Horneck et al., 2011). Enhancing nitrogen levels in the soil requires that farmers increase the quantity of nitrogen inputs through regular addition of organic and inorganic fertilizers. The level of total nitrogen was positively correlated with the amount of organic fertilizer applied (r = 0. 81; p = 0.0006) and the quantity of inorganic fertilizer applied (r = 0.75; p = 0.0007). Similar to what Singh and Mishra (2012) found, total nitrogen had a negative and significant correlation with soil pH.

The level of Phosphorus in the undisturbed parcels was below optimum and also much lower compared to that in the cultivated parcels (Table 3). One possible explanation to this result is that soils in Machakos and Makueni are naturally deficient in Phosphorus (Sketchley et al., 1978). This could be explained by the fact that the hornblende gneisses are highly weathered and therefore poor in nutrients, especially P. Phosphorus plays a critical role in root establishment and other critical plant growth functions and therefore its deficiency can have substantial negative effects on yields (Forth and Ellis, 1997). The improvement of the level of phosphorus in cultivated fields from pristine conditions could be attributed to the addition of phosphorus through inorganic and organic fertilizers. Nevertheless, the level of P in the cultivated plots was generally deficient, considering that the 75th percentile was 19.5 ppm, implying that only 25% of the soil samples were within the optimum band of 20–80 ppm. Phosphorus deficiency is mostly attributed to low pH which makes most of the phosphorus bound into the soil in forms that are not accessible to plants. Further, the correlation between P and OC and RC was positive and significant, indicating that there were low levels of P_o_. High clay content would also lead to limited availability of Phosphorous similar to what Singh et al. (2015) also found.

The three most important micro-nutrients: Copper, Zinc and Boron were tested and averaged at 2.9 ppm, 4.9 ppm, and 0.4 ppm respectively. Only Boron and Copper were deficient and therefore will be discussed in detail. The mean Boron levels were 0.8 ppm, 0.4 ppm and 0.5 ppm in the undisturbed, semi-disturbed and cultivated plots respectively. This is considered deficient given that only values exceeding 1 ppm are adequate (Horneck et al., 2011). Boron is an important micro-nutrient which controls multiple plant functions such as cell wall formation and stability, maintenance of structural and functional integrity of biological membranes, movement of sugar or energy into growing parts of plants and pollination (Brown et al., 2002; Cakmak and Römheld, 1997). Boron also facilitates the uptake of Phosphorus and potassium and limits Aluminium toxicity in low pH soils and occurs in soil solution mainly as undissociated H_3_BO_3_ which dissociates only slightly below pH 9.2 (Goldberg, 1993). Boron was deficient in most samples from both the cultivated and control plots. Cultivation seemed to exacerbate the situation with the percentage of samples with low Boron content growing from 60% in the control samples to 86% in the cultivated samples.

The average level of Copper was 2.0 ppm, 2.1 ppm and 4.3 ppm in the undisturbed, semi-disturbed and cultivated plots respectively. Copper was generally deficient across all land use types given that the critical value is >5 ppm according to Horneck et al. (2011). Copper is an important micro-nutrient that supports lignin synthesis, chlorophyll production, respiration and protein synthesis. It is also a constituent of ascorbic acid, oxydace, phenolace and plastocyanin (Havlin et al., 2010). Copper deficiency may affect yields in cereals due to its effect of grain filling and delayed maturity period (Solberg et al., 1999). Deficiency in Copper in soils from Machakos and Makueni can be explained by the high levels of sand. Bioavailability of copper is also significantly affected by the soil pH.

The mean Organic Carbon was 3% (range: 1.2%–5.1%) and 1.1% (range: 0.41%–5.29%) in the control and cultivated plots respectively. Similarly, the mean reactive carbon in the cultivated plots was 0.07% compared to 0.17% found in the control plots. Over 70% of the samples in the cultivated plots had low OC levels, with similar trends observed in the levels of Reactive Carbon (Table 4). These values are interpreted in reference to the critical values documented in Weeda, 1987. The low levels of organic carbon can be attributed to topological attributes, climatic factors (e.g. temperature and altitude) and farming practices. The Kangundo, Mbooni and Kilungu sites, that were located in high altitude areas with cooler climates had higher levels of organic carbon (F = 33.94; p < 0.01) and reactive carbon (F = 24.48; p < 0.01). Organic carbon is a key determinant of crop productivity since it plays the critical function of nutrient retention, soil aggregation, nourishment of soil organisms, water holding capacity. Through these functions, soil organic carbon is a critical determinant of soil chemical, physical and biological attributes (Chan, 2010).

**Table 4:** Changes in soil quality parameters across land use types.

	Undisturbed land use					Cultivated land use					Semi-disturbed land use				
	% distribution of samples by level			Nutrient Index (NI)	Level of NI	% distribution of samples by level			Nutrient Index (NI)	Level of NI	% distribution of samples by level			Nutrient Index (NI)	Level of NI
	Low	Medium	High			Low	Medium	High			Low	Medium	High		
OC	5.4	45.9	48.6	2.43	**High**	50.6	40.8	8.6	1.58	**Low**	30.6	52.8	16.7	1.86	**Medium**
N	27.0	70.3	2.7	1.76	**Medium**	71.2	28.4	0.4	1.29	**Low**	55.6	44.4	–	1.44	Low
P	83.8	13.5	2.7	1.19	**Low**	32.8	29.6	37.6	2.05	**Medium**	86.1	8.3	2.8	1.14	**Low**
K	–	40.5	59.5	2.59	High	0.6	9.2	90.3	2.90	High	–	44.4	55.6	2.56	High
Ca	32.4	56.8	10.8	1.78	**Medium**	64.9	33.6	1.5	1.37	**Low**	75.0	22.2	2.8	1.28	Low
Mg	–	24.3	75.7	2.76	High	2.3	35.9	61.8	2.60	High	16.7	33.3	50.0	2.33	Medium
B	59.5	27.0	13.5	1.54	**Low**	86.3	12.0	1.7	1.15	**Low**	94.4	5.6	–	1.06	Low
Fe	–	–	100	3.00	High	–	–	100.0	3.00	High	–	–	100.0	3.00	High
Cu	2.7	27.0	70.3	2.68	High	5.5	25.2	69.3	2.64	High	11.1	30.6	58.3	2.47	High
Mn	–	5.4	94.6	2.95	High	6.1	3.8	90.1	2.84	High	11.1	5.6	83.3	2.72	High
Zn	2.7	40.5	56.8	2.54	High	0.8	34.2	65.1	2.64	High	13.9	50.0	36.1	2.22	High

Nutrient Index categories according to Ramamurthy and Bajaj (1969): <1.67 = low, 1.67–2.33 = medium; >2.33 = high. The NI levels in bold shows the Nutrient changes that are critical as a criteria for follow up in subsequent analysis.

### 3.2 Effects of land use on soil quality

Results on the effect of land use of soil fertility parameters are presented on Table 3, Table 4. The last two columns of Table 3 show the percentage changes in soil fertility parameters as a result of (1) conversion of undisturbed land into crop land and (2) conversion of crop land into long term fallows. Results based on the percentage changes were corroborated through an assessment of Nutrient Index categories (Table 4), where a change from a higher NI category to a lower one as land use changes would imply deterioration while improvements would be implied when there is a change from a lower NI category to a higher one. Generally, conversion of land into cropland had both positive and negative effects on soil quality parameters.

Results indicate that agricultural land use was associated with deterioration in the levels of Total Nitrogen, Organic and Reactive carbon, Magnesium, Calcium and Boron. The level of total Organic Carbon and reactive carbon in cultivated plots were 72% and 58% lower than those in undisturbed plots respectively. The Nutrient index changed from high in the undisturbed plots to low in the cultivated plots further confirming the decline. These findings are consistent with Chacon et al. (2015), Khadka et al. (2017) and Kimigo et al. (2008) who all showed that when soil in a previously forested area is cultivated, a high proportion of organic carbon is removed from the site during crop harvest. A similar trend was observed with Nitrogen which declined by 22% as a result of cultivation. Nitrogen is deficient in the study areas, given that the Nitrogen Index level in the undisturbed fields was Medium and further deteriorated to Low in the cultivated fields. The other macro-nutrients that have declined as a result of cultivation were Magnesium, Calcium and Iron. Boron, a critical soil micronutrient was generally low even in the control parcels but deteriorated further in the cultivated fields.

Besides the negative effect of cultivation on soil fertility, it was also noted that cultivation was associated with improvement in some soil quality parameters such as Phosphorus (+73%), Copper (+53%) and Zinc (46%). The improvement in these soil nutrients can be attributed to soil conservation and fertility management practices that involve addition of fertilizers that are rich in the nutrients. Soil conservation practices such as terracing prevent soil erosion and therefore curtails the loss of top soil which is usually responsible for holding most soil nutrients (Gachene et al., 1998).

Another important issue addressed in the current study was the effect of long term fallows on soil quality parameters. Results reveal a positive effect of long term fallows on some soil fertility parameters fertility with the largest improvement reported in total Carbon (16%), and reactive Carbon (11%) and Sodium (8.5%) and Total Nitrogen (7%). Fallowing contributes to improvement of organic carbon through accumulation of litter in fallows and reduced loss of organic matter as a result of minimum activity during the fallow period. Accumulation of organic carbon then is likely to drive recovery of other macro and micro nutrients and general soil fertility.

### 3.3 Explaining nutrient deficiencies: Pearson correlation and CART results

To explain the deficiencies and dynamics in the soil fertility parameters that were identified to be deficient and showing deterioration trends, the study first estimated Pearson Correlation coefficients between these soil parameters and four soil attributes: Organic Carbon, Reactive carbon, pH and Clay content (Table 5). Secondly, the critical factors that can explain variations in the selected deficient nutrients were identified using the Classification and Regression Tree (CART) approach. Correlation coefficient revealed significant relationships between the nutrients that were deficient and the selected soil attributes. Total Nitrogen increased with clay content and organic carbon but declined with soil pH. As expected, phosphorus was less available at lower levels of soil pH and high levels of clay. Improved soil organic carbon and reactive carbon was found to lead to improvement in phosphorus availability. Soil pH was the major variable positively correlated with Calcium. Boron was positively correlated with all the soil attributes included in the analysis implying that the micro-nutrient is influenced by several soil characteristics (Goldberg, 1993). Finally, there was a negative correlation between soil pH and total organic carbon and reactive carbon. At lower pH, there is less microbial activity and therefore low mineralization of organic carbon.

**Table 5:** Pearson correlation coefficients between available nutrients and soil attributes.

	N	P	Potassium	Calcium	Boron	OC (%)	RC (%)
Clay (%)	0.18⁎⁎	−0.25⁎⁎	0.34⁎⁎	0.05^ns^	0.11⁎⁎	0.18⁎⁎	0.11⁎⁎
OC (%)	0.82⁎⁎	−0.04^ns^	−0.01^ns^	0.04^ns^	0.15⁎⁎	1	0.81⁎⁎
RC (%)	0.81⁎⁎	0.12⁎⁎	0.08	0.26⁎⁎	0.33⁎⁎	0.81⁎⁎	1
Soil pH	−0.38⁎⁎	0,30⁎⁎	0.42⁎⁎	0.68⁎⁎	0.43⁎⁎	−0.38⁎⁎	−0.20⁎⁎

⁎⁎ Correlation is significant at 0.01 level, 2-tailed; ns = not significant.

**Fig. 1:**
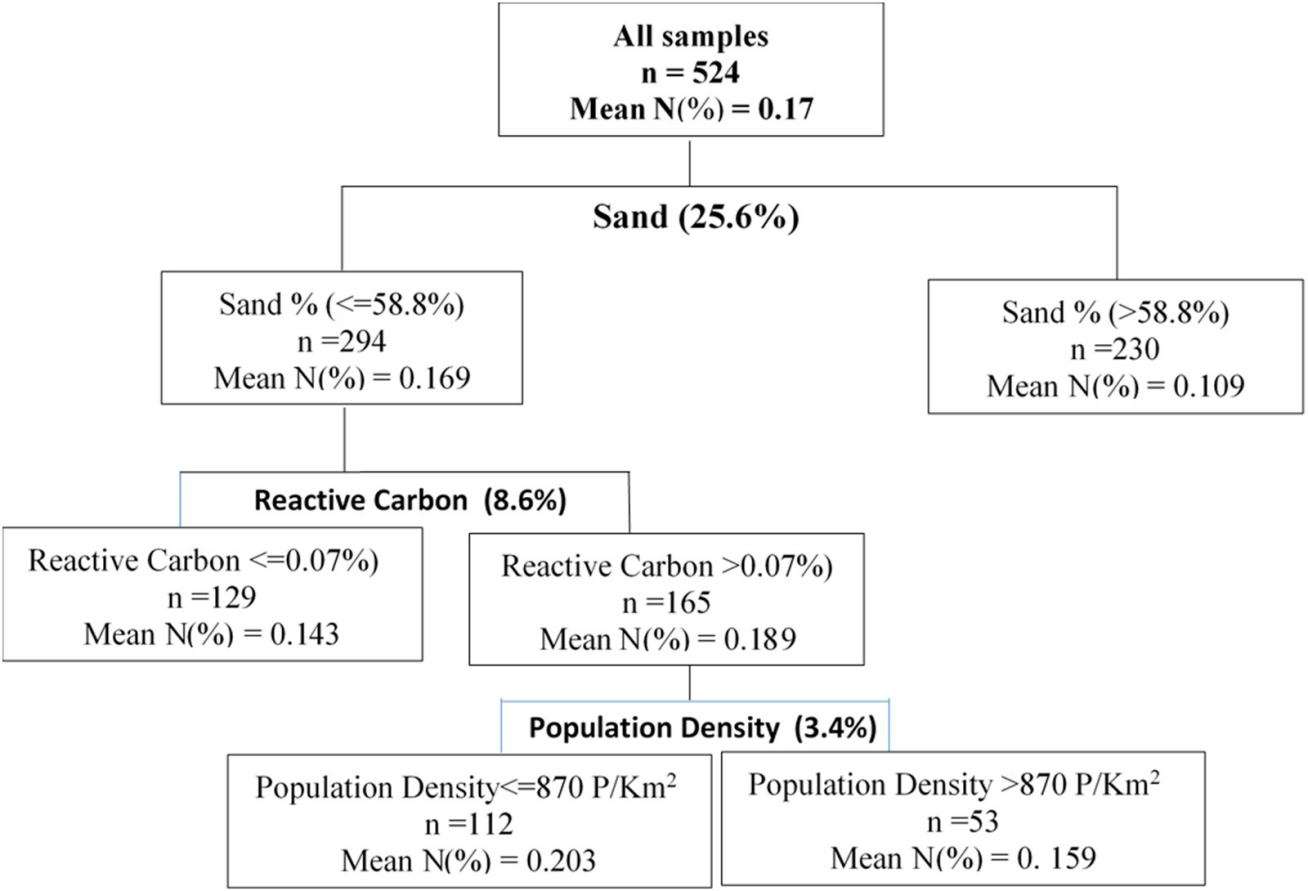
Regression Tree for determinants of nitrogen.

**Fig. 2:**
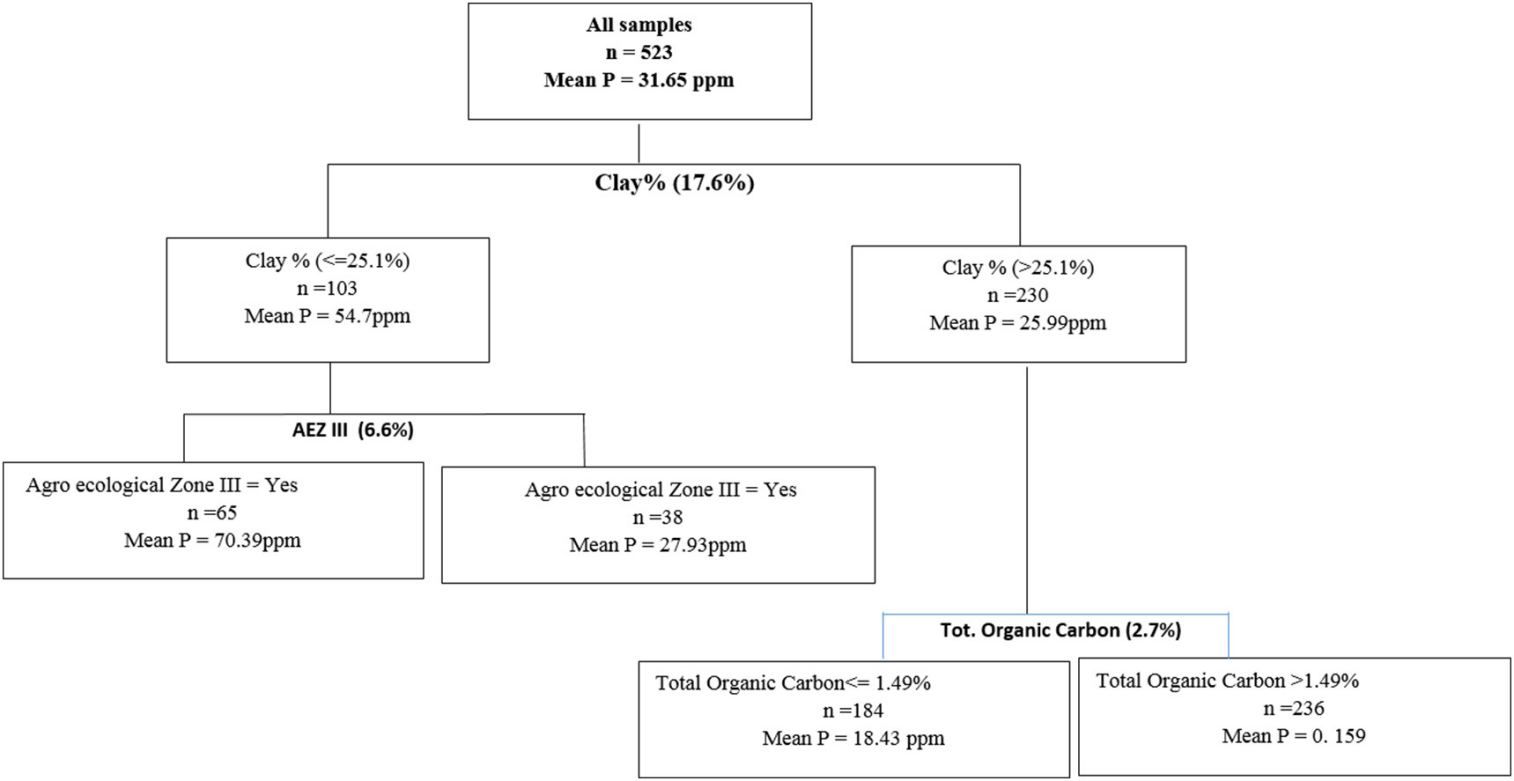
Regression Tree for determinants of phosphorous.

**Fig. 3:**
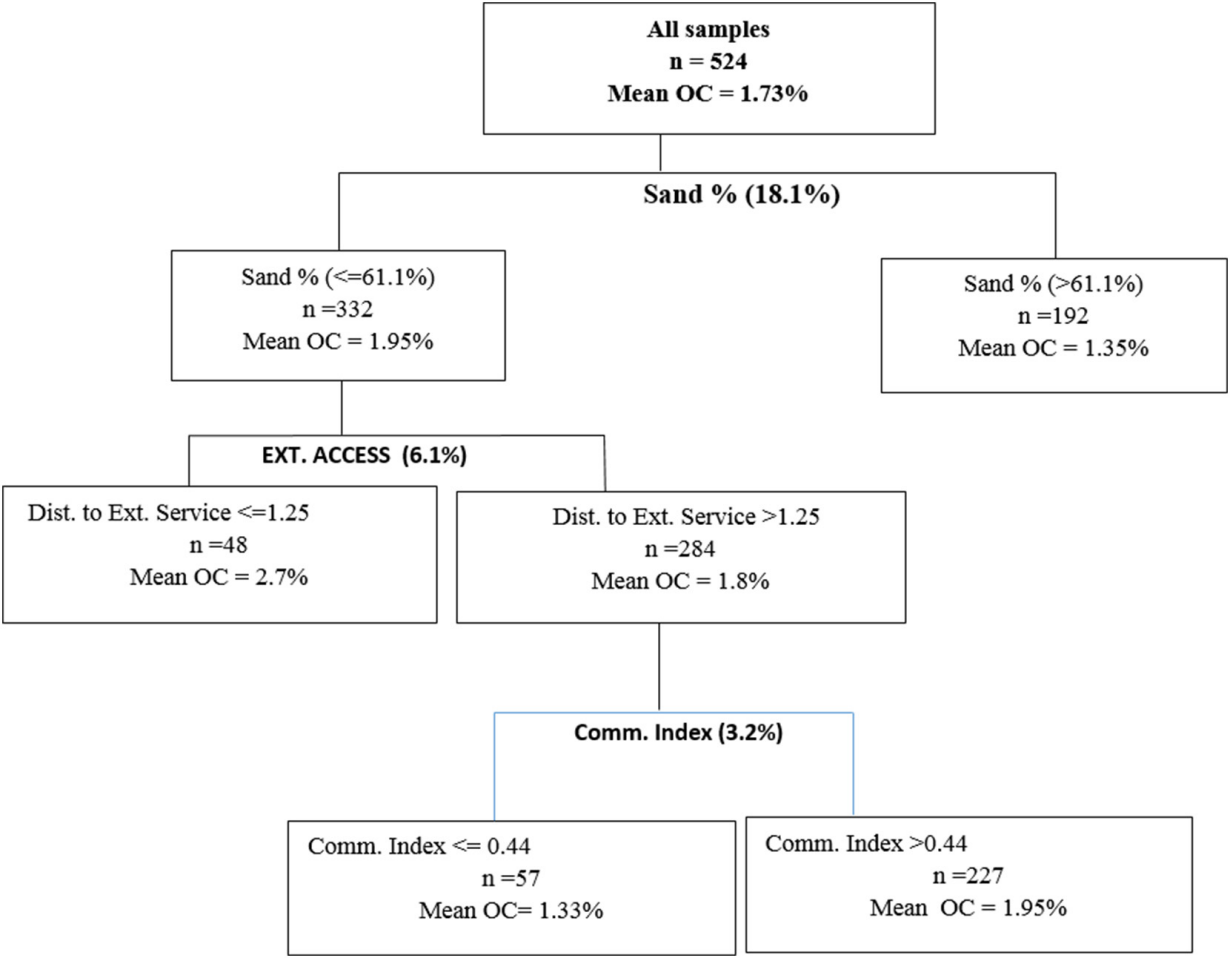
Regression Tree for determinants of organic carbon.

**Fig. 4:**
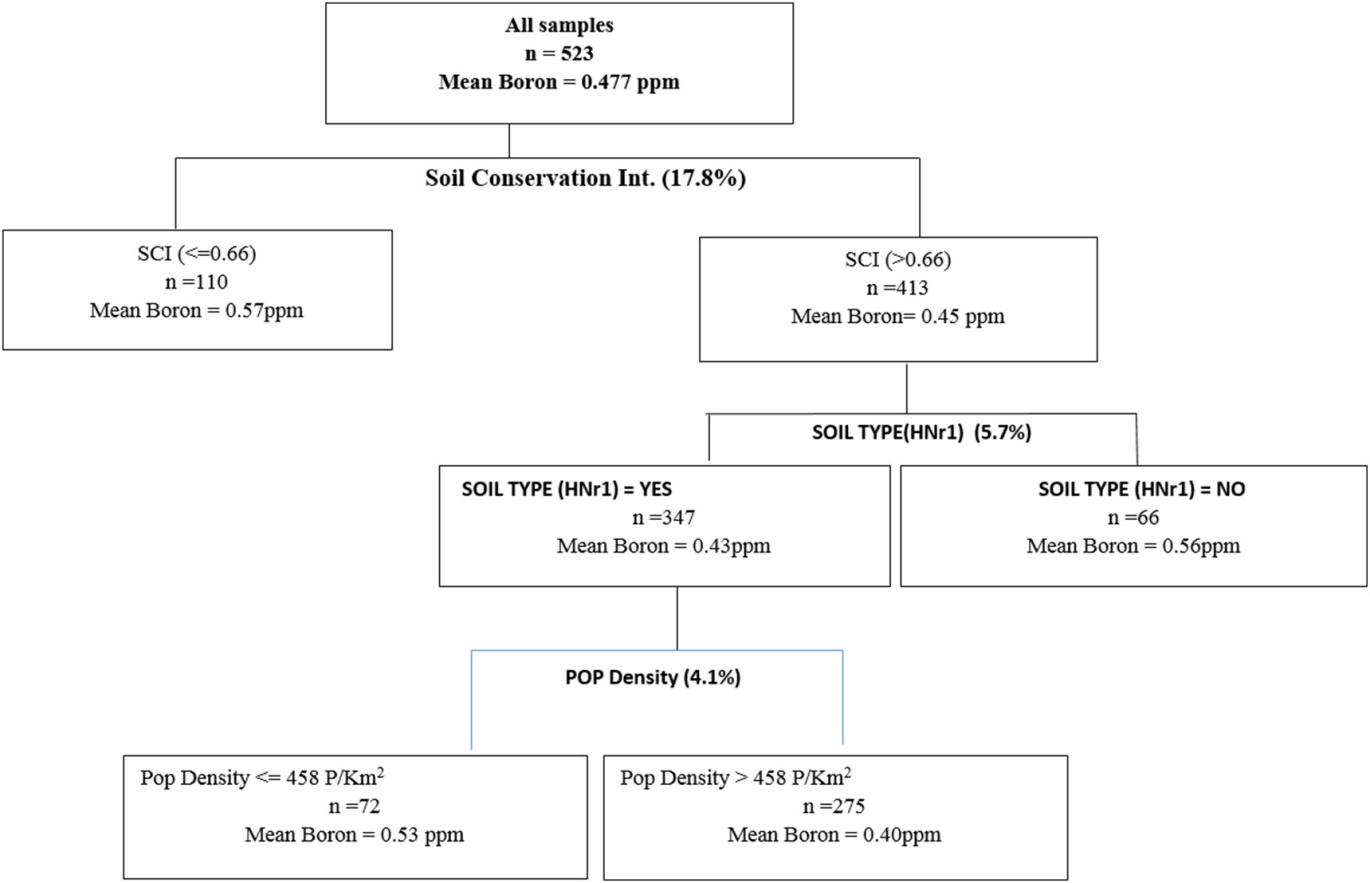
Regression Tree for determinants of variation in boron.

Results from the CART analysis are presented in Fig. 1, Fig. 2, Fig. 3, Fig. 4. The performance test for the CART model yielded satisfactory results with a misclassification rate for the N, P, OC and B trees being 0.042 (Sd = 0.016); 0.075 (Sd =0.032); 0.056 (Sd = 0.024) and 0.098 (Sd = 0.059) respectively. The variation in Total Nitrogen was significantly explained by Sand content (25.6%), Reactive carbon (8.6%) and Population density (3.4%) (Fig. 1). Soil samples with sand content exceeding 58% had approximately 0.11% N contents compared to 0.17% in soils with lower sand content. The node with sand content <58% was further partitioned based on the reactive carbon content, explaining another 8.6% of the variation in Total Nitrogen. The total Nitrogen in soils with reactive carbon exceeding 0.07% was 32% higher than that contained in samples with lower reactive carbon. Clay and C are known to bind most of the N, mostly >95% (Allison, 1973; Stevenson, 1982a; Stevenson, 1982b). The last partitioning was on the node of reactive carbon exceeding 0.07% and was based on the population density which explained 3.4% of the variation in Total N. Plots located in areas with population densities exceeding 877 persons/km^2^ had about 22% lower Total Nitrogen compared to plots in less densely populated areas. Population density is a proxy for agricultural intensification characterized by intensive cultivation and reduced fallows which could explain the reduction in Total Nitrogen content.

The Phosphorus regression tree (Fig. 2) was split based on Clay content, Agro-Ecological Zone and Total Organic Carbon which explained 17.6%, 6.6% and 2.7% of the variation respectively. Soils with clay content >25% had a mean P of 25.5 ppm which was slightly over 50% less than that contained in samples with <25%. Soils drawn from plots found in Agro-Ecological Zone III had three times more plant available phosphorus compared to plots located in other Agro-Ecological Zones (II and IV). Farms in AEZ III were mainly found in high altitude areas of Mbooni, Kanzalu and Kilungu. The soil types found in these areas were the MQ1/DF and HNr1/DF. Given the high altitude, these areas also happen to be colder and with higher precipitation. The node of soils with >25% clay content were further split based on the organic matter content, which explained 2.7% of the variation in P. Soils with organic matter content that was >1.4% had almost double P content, indicating that most of the P is in the inorganic form (P_i_) which can be attributed to the parent material found in the area.

The most important variable that explained the variation in Organic Carbon was soil texture explaining 18.1% of the variation in the OC content (Fig. 3). The node with <61% Sand content was further split based on access to extension service providers, which explained 6.1% of the variation in the OC. Farmers who were located <1.25 km from the extension service providers had more OC levels compared to those who were located further from the service providers. Access to extension services is an indicator of expert advice on soil fertility management which could translate to higher levels of OC. The node of farmers who were located further from the extension service providers was then split based on commercialization index which explained an additional 3.2% of the variation in OC. Farmers who were more commercially oriented had 31% more OC compared to subsistence farmers. Commercial farmers have more incentives to invest in soil fertility management practices which they can easily afford based on the returns they generate from commercial farming.

The last regression tree was done for Boron and presented in Fig. 4. The variation in the level of Boron was explained by the intensity of soil conservation, soil type and population density. Farms with high intensity of soil conservation were associated with lower levels of Boron. Given that soils in the study areas were generally deficient in Boron (even in the undisturbed plots), soil conservation does not seem to improve the situation. Further, soils from farms with high conservation intensity were split into more nodes based on soil type. The soil type HNr1 which is found mainly in Kanzalu and Iveti Hills contained higher levels of Boron than any other soil types. The soil type explained 5.7% of the variation in the quantity of Boron. Finally, the node with soil types HNr1 was split based on the population density which explained 4.1% of the variation in Boron. Soils from areas with population density exceeding 458 persons/km^2^ contained an average 0.42 ppm compared to 0.5 ppm found in soils drawn from less densely populated areas.

## 4 Conclusions and policy implications

This study was motivated by the need for a clear understanding on how cultivation has affected soil quality in densely populated areas, which specific soil quality parameters are affected by cultivation and what are the exogenous and endogenous drivers of the changes in the individual soil quality parameters. This is importance considering that densely populated areas are facing pressure to meet the growing food demand in a sustainable way. The study applied descriptive statistics and the Nutrient Index approach to identify the soil nutrients that were deficient in the soils drawn from three land use types (Undisturbed, Semi-disturbed and Cultivated) in Machakos and Makueni counties, Kenya.

The study finds deficiencies in Total Nitrogen, Phosphorus, Organic Carbon, Reactive Carbon and Boron. Results also indicate that soils in the study area are naturally deficient in Phosphorus and Boron. Further, agricultural land use was found to have negatively influenced critical fertility parameters such as Nitrogen, Organic Carbon, Reactive Carbon and Calcium. The variations in these nutrients were found to be determined by soil texture, the level of soil organic carbon, access to extension services, commercialization index, soil type, agro-ecological zones and population density. Long term fallowing was found to be associated with recovery of soil fertility with improvement in Phosphorus, Total Nitrogen, Sodium, Magnesium, Organic Carbon and Reactive Carbon. A novel finding that can be relevant for the densely populated areas which are constantly facing pressure from intensive cultivation is that better management practices can safeguard crop land from imminent deterioration regardless of the period under cultivation. The current study didn't find any proof that farm lands that were cultivated for longer periods were worse off so long as farmers engaged in practices that restored organic matter and prevented soil erosion. Innovations in soil and water conservation can delay the threshold after which crop land would enter into a phase indefinite deterioration.

The study concludes that although some soil nutrients are deficient in the land in pristine conditions due to the attributes of parent materials and environmental factors, cultivation in densely populated areas has resulted to deterioration in soil quality. This deterioration can be linked to endogenous factors such as the agricultural practices, soil physical, chemical and biological attributes and exogenous drivers such as environmental factors that define agro-ecological zones (temperature and rainfall) and institutional factors (access to extension services). Long term fallowing was found to be a strategy that can effectively restore some of the lost soil nutrients. Nevertheless, there is need for incentives for organic fertilizer application and delimited and targeted inorganic fertilizer application. Inorganic fertilizers should be applied with caution, avoiding the application of acidic fertilizers in soils with low pH. Blending of commercial fertilizer brands already in the markets with critical micro-nutrients that are naturally deficient in soils is encouraged. Practices that facilitate soil organic matter build-up such as organic fertilizer application and incorporation of crop residues should be encouraged to boost organic carbon which is a key determinant of soil fertility which in return determines the availability of all major soil nutrients.
